# Evaluation of the Efficacy of Topical Tetracycline in Enhancing the Effect of Narrow Band UVB against Vitiligo: A Double-Blind, Randomized, Placebo-Controlled Clinical Trial

**DOI:** 10.1155/2014/472546

**Published:** 2014-02-09

**Authors:** Amir Kalafi, Farideh Jowkar

**Affiliations:** ^1^Student Research Committee, Molecular Dermatology Research Center, Department of Dermatology, Shiraz University of Medical Sciences, Shiraz, Iran; ^2^Molecular Dermatology Research Center, Department of Dermatology, Shiraz University of Medical Sciences, Shiraz, Iran

## Abstract

*Background*. Vitiligo is a pigmentary disorder characterized by depigmented macules due to absence of melanocytes. Increased levels of tumor necrosis factor alpha and interleukin-1 in the epidermis of lesions may play a role in keratinocyte apoptosis and less production of melanogenic cytokines. Tetracyclines reduce production of tumor necrosis factor alpha and interleukin-1. *Objective*. To evaluate the effect of topical tetracycline on vitiligo patients on phototherapy. *Methods*. Thirty cases of generalized stable vitiligo were chosen randomly and pigmentation of two assigned lesions on right and left sides (same size and location) was determined by vitiligo area severity index, and medication and placebo were randomly assigned to be applied twice daily on either right or left side, respectively. Images were taken of the lesions at the end of the 4th, 8th, and 12th weeks and pigmentations were compared to baseline using aforementioned index. The patients also took narrow band ultraviolet B two to three times a week. *Results*. Mean pigmentation, based on vitiligo area severity index, changed significantly from 90.1667 to 86.6667 (*P* = 0.026) and on placebo side from 89.6667 to 86.8333 (*P* = 0.026). There was no significant difference between medication and placebo sides in terms of pigmentation (*P* = 0.566). *Conclusions*. No significant difference in improving repigmentation between medication and placebo sides was seen.

## 1. Introduction

Vitiligo is an acquired pigmentary disorder that presents with depigmented macules or patches that is due to absence of epidermal melanocytes.

Men and women are equally affected. At least 30% of patients have positive family history of vitiligo [1].

In most cases, the disorder begins between 10 and 30 years [2].

Generalized form (symmetric involvement of more than 20% of body surface area) is the most common type.

Antibodies to melanocytes are suggested as one possible cause of vitiligo [1].

In another hypothesis, expression of epidermal cytokines in lesions may be different when compared to normal skin. Keratinocyte-derived cytokines such as tumor necrosis factor-alpha (TNF-alpha) are thought to affect melanocyte survival and function [3].

There are increased levels of TNF-alpha and interleukin-1 (IL-1) in vitiligo lesions compared to normal skin. These cytokines seem to be able to destroy melanocytes [4].

Keratinocytes are involved in melanocyte homeostasis, so any change in keratinocytes may cause melanocyte dysfunction. High levels of TNF-alpha may play a role in keratinocyte apoptosis, which leads to less production of melanogenic cytokines and therefore melanocyte loss [5]. So, TNF-alpha is considered as a complex mediator that regulates melanocyte destruction [6]. Also TNF-alpha genotype polymorphism (GA genotype was significantly higher in vitiligo patients) has been associated with an increased risk of vitiligo in Saudi patients. This study indicated a critical role for this proinflammatory cytokine in pathogenesis of vitiligo [7].

This polymorphism (TNF-alpha 308 GA genotype) has also been possible in Mexican population and is believed to increase plasma levels of TNF-alpha [8].

Some resistant generalized cases were associated with high skin levels of TNF-alpha, and improvement with TNF-alpha inhibitors like etanercept is reported [9].

Tetracyclines reduce production of TNF-alpha and IL-1 [10] and are capable of regulating inflammatory cytokines and inhibiting leukocyte chemotaxis and also have antioxidative properties [11]. Narrow band ultraviolet B (NB-UVB) is a good option in the treatment of generalized vitiligo [2]. We decided to evaluate the efficacy of topical tetracycline ointment on vitiligo patients who were also receiving NB-UVB. It is necessary to mention that topical tetracycline has been used for the treatment of acne vulgaris (for 2 months, effective by inhibiting inflammation due to normally commensal organism) [12] and some superficial skin infections [10], but it has never been tested for vitiligo (no related article has been published yet). So based on the fact that systemic tetracyclines reduce production of TNF-alpha and IL-1 and are capable of regulating proinflammatory cytokines and inhibiting leukocyte chemotaxis and also have antioxidative properties, this study assumes that topical tetracycline ointment might reduce these cytokines and has less side effects than systemic agents.

This study was done for the first time (to our knowledge).

## 2. Methods

### 2.1. Patient Selection

Our study was double-blind, randomized, placebo-controlled clinical trial. The patients were randomly selected from the Outpatient Dermatology Clinic of Faghihi Hospital (we randomly selected patients via choosing some numbers among patient receipt numbers for phototherapy in phototherapy center each day). Patients with generalized, stable vitiligo (nonprogressive or no new lesions in the last 3 months) were included in our study.

Pregnant or lactating cases, children less than 8 years old, cases of photodermatoses or cutaneous malignancy, or those who had used other medications for vitiligo in the last 3 months were excluded.

### 2.2. Study Protocol

Ethical committee of Shiraz University of Medical Sciences approved the study. Informed consent was signed by each patient.

A questionnaire about family history of vitiligo, associated diabetes or thyroid problems, demographic information, medications taken by patient, number of phototherapies prior to study, distribution of lesions, status of disease (stable or progressive), and side effects during trial was filled out.

Vitiligo cases were randomly told to apply tetracycline ointment twice daily on the right or left side assigned lesion and placebo (vaseline colored yellow by artificial dyes used in cooking) twice daily on the other side lesion (almost same size and position). All patients were told to apply ointments only at night if they receive phototherapy in the morning, since tetracycline ointment may induce photosensitivity in the patients if applied in the morning and then get phototherapy. Patients also were receiving narrow band ultraviolet B (NB-UVB) phototherapy twice or thrice weekly. Each case was evaluated and visited at the end of the 4th, 8th, and 12th week.

A third person assigned A and B to both ointments, so both patient and physician were blinded to treatment and placebo.

### 2.3. Assessments

Pigmentation of selected lesions on both sides was determined based on vitiligo area severity index (VASI) prior to study and photos were taken of both sides (baseline, at the end of 4th, 8th, and 12th weeks).

Images were compared to baseline based on VASI.

*VASI Score.* Consider the following 100% = depigmentation, 90% = specks of pigment, 75% = depigmented areas more than the pigmented areas, 50% = equal pigmented and depigmented areas, 25% = pigmented areas more than depigmented area, and 10% = specks of depigmentation. 

Cases were told to report any side effect whether from phototherapy or ointments.

### 2.4. Statistical Methods

Paired *t*-test and Wilcoxon signed-rank test were used for statistical analysis (with SPSS 15 software).

All results were significant if *P* values were 0.05 or less. We chose sample size of 30, based on a study (topical tacrolimus for vitiligo therapy, by Grimes PE, 2004 with 23 patients) as mentioned in Section 4 (refer to 14 in reference), with @ = 0.05 and power 80%.

## 3. Results

### 3.1. Patient Population

**Figure 1:**
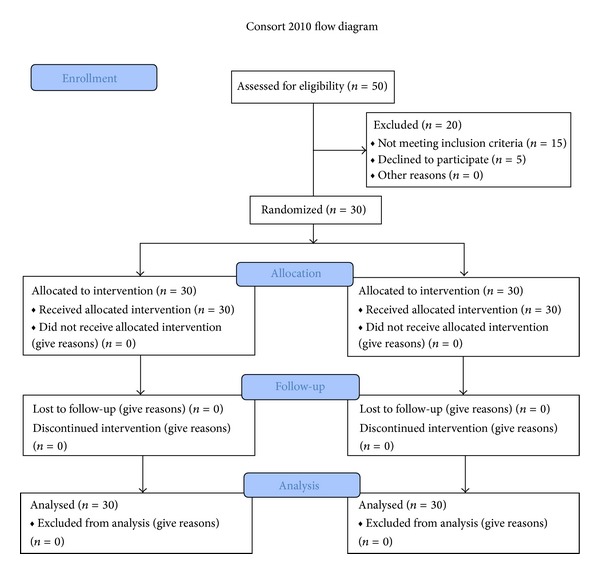


There were 30 cases (17 (56.7%) females and 13 (43.3%) males), with generalized stable vitiligo, aged from 11 to 66 years, enrolled in our study. Cases were assigned randomly to apply treatment and placebo ointments to right or left side lesions (similar location and size). The patients also were receiving NB-UVB, 2-3 times weekly. All cases completed the 12-week study. The process of patient selection is shown Figure 1.

### 3.2. Treatment Efficacy

Results showed that 23.3% of cases had positive family history of vitiligo, 16.6% were diabetic, and 13.3% had thyroid problem. Sites tested during this study and number of cases were as follows: elbow 12, knee 2, leg 5, wrist 2, neck 1, arm 2, forearm 3, abdomen 1, and chest 2. In Table 1, we have reported pigmentation (according to VASI) before and after medication and placebo. Since most changes in the pigmentation were observed at the end of the 12th week, we only have reported the final pigmentation in this study (Table 1). Mean pigmentation before and after trial on both sides (Table 2) and the difference between two sides are reported (Table 3) in the tables.

There was a significant change in medication side in terms of pigmentation compared to baseline (*P* = 0.026).

Also similar change in the placebo side was observed (*P* = 0.026).

There was no significant difference between medication and placebo sides in terms of pigmentation (*P* = 0.566).

In summary in the five cases (16.6%), improvement in pigmentation was observed on medication side compared to placebo side. Similarly, 16.6% of cases showed better repigmentation on placebo side compared to medication side.

### 3.3. Safety and Tolerability

A few patients complained of stinging at the site of treatment, but in general, no adverse cutaneous effect reported.

## 4. Discussion

This study evaluates the efficacy of topical tetracycline ointment in enhancing the effect of NB-UVB (narrow band ultraviolet B) phototherapy against generalized, stable vitiligo (nonprogressive or no new lesions in the last 3 months), after the end of the 4th, 8th, and 12th week.

Since we know systemic tetracyclines can reduce production of proinflammatory cytokines such as TNF-alpha and IL-1 [10], and these cytokines are high in the epidermis of vitiliginous skin [3], may cause damage to melanocytes, inhibit melanocyte stem cells differentiation, and induce various apoptotic pathways [13], we decided to test topical tetracycline that might be beneficial against vitiligo and has less side effects than systemic agents. There are some studies that report successful therapy of some inflammatory skin disease with TNF-alpha reducing agents. An ointment like tacrolimus (that is effective in the treatment of vitiligo) decreases TNF-alpha expression in epidermis [14] and inhibits the activation of the nuclear factor for activated T cells (NFAT) [15].

Two cases of refractory generalized vitiligo were reported by Kim et al. [9] to improve with etanercept (TNF-alpha inhibitor) taken subcutaneously weekly. The authors suggested that high vitiligo disease activity score is related to high TNF-alpha level in the epidermis.

Vitiliginous lesions of a patient (reported by Lv et al. [13]) disappeared following infliximab (TNF-alpha inhibitor) IV. The authors hypothesized that TNF-alpha inhibits melanocyte stem cells differentiation; thus TNF-alpha inhibitors may serve as an effective therapy for vitiligo.

In a study by Laddha et al. [16], female patients and active vitiligo or generalized vitiligo patients had higher levels of TNF-alpha. Also TNF-alpha polymorphism correlated with disease progression. In this study it is mentioned and concluded that TNF-alpha inhibits melanocyte production. This cytokine has an important role in many autoimmune diseases. Thus again there is some emphasis toward potential role of this cytokine in the pathogenesis of vitiligo. So, it is concluded (from this study) that targeting this cytokine may serve as an additional treatment against vitiligo.

On the other hand, in a study by AlGhamdi et al. [17], in 6 cases of generalized stable vitiligo, TNF-alpha inhibitors like infliximab, etanercept, and adalimumab were tested. Again, TNF-alpha was supposed to inhibit melanocyte stem cell differentiation and destruct them via some apoptotic processes. Digital images were taken before and after treatment and compared, but no significant repigmentation and efficacy were observed, but medications were well tolerated.

In a case report, the use of etanercept (TNF-alpha inhibitor) subcutaneously improved a patient affected by both psoriasis and vitiligo, although there was mild improvement of vitiligo. Thus in this case report, the role of TNF-alpha seems probable in vitiligo [18]. Some studies like Exarchou et al. [19] reported some autoimmune diseases, like vitiligo, development after TNF-alpha inhibitor therapy, but vitiligo developed only in one patient. This study was done on 252 cases. Levels of Fas ligand are also shown to be higher in depigmented epidermis in 10 patients by Kim et al. [20].

Infliximab (TNF-alpha inhibitor, IV) improves not only ankylosing spondylitis, but also the vitiligo, probably due to anti-TNF-alpha activity blockage. This was a case report [21].

So, based on the aforementioned studies, some are in favor, and some are against it. It is sensible to do larger studies on TNF-alpha inhibiting agents since there is no definite cure or safe therapy for vitiligo patients so far. It is suggested that topical agent that may reduce TNF-alpha in the epidermis (like tetracycline) is evaluated further in the treatment of vitiligo, since it is safe and not as expensive as TNF-alpha blocking agents.

Limitation in this study is the short duration of clinical trial and the limited number of patients, although based on a textbook [4], 2-3 months are needed for evaluating a topical therapy against vitiligo. This study on the efficacy of topical tetracycline ointment in enhancing the effect of NB-UVB phototherapy against generalized, stable vitiligo showed no significant improvement in pigmentation in the lesions on topical tetracycline side, compared to placebo side. No related article has been published yet on the use of the topical tetracycline ointment against generalized, stable vitiligo.

## 5. Conclusions

Our study on 30 cases of generalized vitiligo showed no significant improvement in pigmentation in the lesions on topical tetracycline side, compared to placebo sides. Although theoretically the application of tetracycline ointment might be helpful, reducing of TNF-alpha in the epidermis, other pathogenic events like autoantibodies against melanocytes or oxidative stress may be stronger. Since this study on topical tetracycline was done for the first time (to our knowledge), we recommend further and larger studies be done on the agents that reduce expression of TNF-alpha in the epidermis of vitiliginous skin, preferably topical in order to lessen side effects.

## Figures and Tables

**Table 1:** Pigmentation of lesions, based on VASI (baseline, end of trial).

Patient number	Pig. (VASI) before Med.	Pig. (VASI) before placebo	VASI 12th week Med.	VASI 12th week placebo	Phototherapy sessions before study	Site of lesion
1	100	100	100	90	1	elbow
2	100	100	100	100	8	elbow
3	75	75	75	50	170	elbow
4	100	100	90	100	150	leg
5	100	100	100	100	51	elbow
6	100	100	100	90	24	leg
7	100	100	100	100	17	leg
8	100	100	100	90	70	neck
9	75	100	75	100	34	leg
10	75	50	50	50	60	knee
11	50	50	50	50	45	elbow
12	90	90	75	90	48	knee
13	90	90	75	75	100	leg
14	100	100	100	100	22	elbow
15	100	100	100	100	15	forearm
16	100	100	100	100	150	forearm
17	90	90	90	90	20	wrist
18	90	100	75	100	32	forearm
19	100	100	100	100	1	elbow
20	75	75	75	75	3	elbow
21	90	100	90	100	7	wrist
22	90	90	90	90	115	abdomen
23	75	75	75	75	130	elbow
24	100	75	90	75	60	elbow
25	75	75	75	75	30	elbow
26	100	100	100	100	43	elbow
27	90	90	90	90	22	chest
28	100	100	100	100	42	arm
29	100	100	100	100	12	arm
30	75	50	75	50	36	chest

**Table 2:** Mean scores of pigmentation based on VASI (medication and placebo sides).

	Mean pigmentation	*N*	Std. deviation
Before med.	90.1667	30	12.69560
After med.	86.6667	30	14.87496
Before placebo	89.6667	30	15.97052
After placebo	86.8333	30	17.24486

**Table 3:** Difference in mean pigmentation between medication and placebo sides.

	Mean (depig.-repig.)	*N*	Std. deviation
Diff. med. side	3.5000	30	6.65444
Diff. placebo side	2.8334	30	5.21106
